# A Case of Cauliflower

**Published:** 1871-04

**Authors:** E. R. Willard

**Affiliations:** Wilmington, Illinois


### A CASE OF CAULIFLOWER.

BY E. R. WILLARD, M.D., Wilmington, Illinois.

Mr. C. called at my office on the 9th of October, 1868, to consult me in the case of his wife. He said she had been unwell almost constantly the past twelve months, and more or less so for eighteen months; that she was subject to frequent fainting spells when attempting to walk about the house.

I requested him to bring his wife to the office, that I might ascertain the cause of the hemorrhage. This he claimed to be impossible, in her present state of health, and the distance from town ten miles, and I must send her some medicine, which I consented to do, with the understanding that I was to see her if she was not relieved with the treatment prescribed.

From this I heard nothing more of the patient until I was summoned to see her, on March 12th, 1869.

I found the patient about 40 years of age, nervous, sanguine temperament, and mother of several children, in bed, so anæmic as not to show the least color of blood in the lips, with some anasarca, and much emaciation, pulse 120, tongue clean, bowels constipated, appetite rather poor. She stated that she had been subject to frequent attacks of hemorrhage the past eighteen months, that lasted for two weeks at a time; between these attacks she would have a profuse watery or bloody-water discharge constantly.

On making an examination, per vaginum, a soft, irregular tumor was found blocking up the passage, and extending to the vulva, which bled profusely on attempting to pass my finger around it, but without any sensation of pain. The tumor seemed to have distended the vagina to its utmost capacity laterally, while its insertion was found to embrace the whole of the cervix uteri, extending up the entire length of the neck, except a narrow point anteriorly.

By ocular examination the excresence at the vulvæ exhibited the deep red color and nodular appearance of a fully ripe strawberry. I diagnosed cauliflower, and after explaining to the patient the nature of the case, and the uncertainty of a favorable issue, I proposed the removal of the growth by amputation of the neck of the uterus, as the only measure calculated to produce a permanent cure. Therefore, on the following day, in consultation with Dr. W. R. Fox, now of SanLeandro, California, who fully coincided in the opinion, that amputation of the neck, as high up as possible, would promise best for the removal of the greater part of the morbid tissue, and, in his opinion, would afford the only chance of safety to the patient, amputation was performed.

Among the different operations recommended and practised for amputation of the neck, as by the écraseur, the scissors, knife, or the wire heated by the electric current, I chose the former, as the most manageable, both as to hemorrhage and subsequent treatment, this was applied, and the neck amputated as high up as possible without endangering the peritoneal cavity. There was very little hemorrhage following the operation, owing in part, no doubt, to the shock upon the system, which in this case was great. The oozing was stopped by free injections of the persulphate of iron, pain relieved by morphine, and the system well-sustained by stimulants, tonics, and beef-essence. In twenty days from the operation the wound on the right side of the cervix appeared to be well covered with healthy granulations, while that upon the left section of the granulating surface presented that peculiar nodulated, strawberry appearance of "proud flesh," shooting up from the surface to the extent of half an inch in breadth to an inch in length, having all the peculiarities of the former growth.

This part was freely cauterized with chromic acid, and the application repeated about once every four weeks up to the end of November. After which the entire wound healed over, without any further development of morbid tissue, exhibiting a smooth, even surface. Between the times of using the cautery, an injection of a weak solution of chromic acid, alternated with that of permanganate of potash, was used occasionally, to correct the fetor.

The patient who had presented such an anæmic, pale, cadaveric, and emaciated appearance at the commencement, now began to regain her former plumpness and cheerfulness.

April 25th, 1870, on making an examination with the speculum, the cicatrix presented a smooth, healthy appearance, no remnant of the epithelioma could be seen, and the general health of the patient was quite good, insomuch, that she was able to attend to all of her household duties. At this writing, March, 1871, she is still enjoying the best of health.

